# CoronaVac and ChAdOx1 Vaccination and Gamma Infection Elicited Neutralizing Antibodies against the SARS-CoV-2 Delta Variant

**DOI:** 10.3390/v14020305

**Published:** 2022-02-01

**Authors:** Marcilio Jorge Fumagalli, Luiza Antunes Castro-Jorge, William Marciel de Souza, Patrick Orestes de Azevedo, Alana Witt Hansen, Ricardo Tostes Gazzinelli, Benedito Antônio Lopes da Fonseca, Fernando Rosado Spilki, Luiz Tadeu Moraes Figueiredo

**Affiliations:** 1 Virology Research Center, Ribeirão Preto Medical School, University of São Paulo, Ribeirão Preto, São Paulo 14049-900, Brazil; luizacastro@gmail.com (L.A.C.-J.); baldfons@fmrp.usp.br (B.A.L.d.F.); ltmfigue@fmrp.usp.br (L.T.M.F.); 2 World Reference Center for Emerging Viruses and Arboviruses, Department of Microbiology and Immunology, University of Texas Medical Branch, Galveston, TX 77555-1019, USA; wmarciel@hotmail.com; 3 Platform of Translational Medicine, Oswaldo Cruz Foundation, Ribeirão Preto Medical School, University of São Paulo, Ribeirão Preto, São Paulo 14049-900, Brazil; patrickoazevedo@gmail.com (P.O.d.A.); ricardo.gazzinelli@umassmed.edu (R.T.G.); 4 Molecular Microbiology Laboratory, Healthy Science Institute, Feevale University, Novo Hamburgo, Novo Hamburgo 93525-075, Brazil; awitt.hansen@gmail.com (A.W.H.); fernandors@feevale.br (F.R.S.)

**Keywords:** SARS-CoV-2, Delta variant, Gamma variant, CoronaVac, ChAdOx1, vaccination, convalescence, antibodies, neutralization

## Abstract

The emergence of new SARS-CoV-2 variants represents a constant threat to world public health. The SARS-CoV-2 Delta variant was identified in late 2020 in India; since then, it has spread to many other countries, replacing other predominant lineages and raising concerns about vaccination efficiency. We evaluated the sensitivity of the Delta variant to antibodies elicited by COVID-19 vaccinated (CoronaVac and ChAdOx1) and convalescent individuals previously infected by earlier lineages and by the Gamma variant. No reduction in the neutralizing efficacy of the Delta variant was observed when compared to B lineage and a reduced neutralization was observed for the Gamma variant. Our results indicate that neutralization of the Delta variant is not compromised in individuals vaccinated by CoronaVac or ChAdOx1; however, a reduction in neutralization efficacy is expected for individuals infected by the Gamma variant, highlighting the importance of continuous vaccination even for previously infected individuals.

## 1. Introduction

The severe acute respiratory syndrome coronavirus-2 (SARS-CoV-2) is the causative agent of the coronavirus disease 2019 (COVID-19) pandemic. SARS-CoV-2 is evolutionary related to other life-threatening respiratory viruses, including MERS-CoV and SARS-CoV, both from the *Betacoronavirus* genus and *Coronaviridae* family [1]. As of 28 December 2021, more than 275 million cases with approximately 5.3 million deaths related to COVID-19 have been reported in the world [2]. As counter-measures to prevent COVID-19, multiple vaccines based on different platforms have been developed, showing varying degrees of efficacy and acceptable safety [3]. However, the continuous spread and evolution of SARS-CoV-2 lead to the emergence of variants of concerns (VOCs), representing a constant threat and raising concerns about the vaccine’s efficiency.

The emergence of new VOCs is associated with mutations in the viral spike (S) gene, and some studies have suggested increased transmissibility, hospitalization rate, mortality, breakthrough infection, and neutralization resistance to vaccine- or natural- acquired antibodies [4,5,6,7,8]. The current VOCs have been reported between the late 2020 and late 2021 in the UK (PANGO lineage: B.1.1.7, WHO: Alpha), South Africa (PANGO lineage: B.1.351, WHO: Beta), Brazil (PANGO lineage: P.1, WHO: Gamma), India (PANGO lineage: B.1.617.2, WHO: Delta), and South Africa (PANGO lineage: B.1.1.529, WHO: Omicron), and currently have been detected in 175, 119, 88, 182, and 116 countries, respectively [9]. To determine the efficiency of neutralizing antibodies capacity elicited by convalescent and vaccinated individuals against the Delta variant, we evaluated neutralizing activity of serum samples from CoronaVac (Sinovac) and ChAdOx1 (AstraZeneca) vaccinated persons, and convalescent serum from individuals infected during the early pandemic (May 2020) and convalescent Gamma infected individuals. 

## 2. Materials and Methods

### 2.1. Ethics Statement

The procedures involving human subjects followed Brazilian regulations and international ethical standards that were approved by the Research Ethics Committee of the Ribeirão Preto Medical School at the University of São Paulo (approval number: 2021/4.545.390) and Institutional Ethical Review Board of the Feevale University (approval number: 33202820.7.1001.5348), Brazil. All participants provided written informed consent.

### 2.2. Human Subjects

Serum samples were collected from volunteers vaccinated with CoronaVac (Sinovac Biotech) or ChAdOx1 (AstraZeneca) vaccines and COVID-19 convalescent individuals. A vaccinated group of volunteers was recruited at Clinics Hospital of Ribeirão Preto, São Paulo State, and the infected group at emergency care units of Campo Bom and Rolante municipalities in the Rio Grande do Sul state, both in Brazil. The eligibility criteria for the vaccinated group included: being at least 18 years old, having no history of COVID-19, and at least 14 days after the second dose of vaccination. The eligibility criteria for the non-VOCs convalescent group included confirmation of infection by a previously described real-time polymerase chain reaction (RT-PCR) specific for SARS-CoV-2 [10]. Lastly, the eligibility criteria for the Gamma convalescent group included confirmation of Gamma variant infection by a specific previously described RT-PCR [11]. Serum samples from both convalescent groups were collected at least 19 days after the confirmatory infection test and disease resolution.

### 2.3. Epidemiological Data 

The data of daily confirmed cases, death rates caused by COVD-19, and vaccination rate in Brazil (1 January to 31 December 2021) were obtained from Our World in Data database (https://ourworldindata.org/coronavirus (accessed on 15 December 2021)) [12]. The frequency of SARS-CoV-2 Alpha, Gamma, and Delta variants was determined based on the annotation by Phylogenetic Assignment of Named Global Outbreak (PANGO), download from the GISAID database (https://www.gisaid.org (accessed on 15 December 2021)). The data visualization was plotted using GraphPad Prism 8.0.2.

### 2.4. Viral Propagation and Cell Culture

SARS-CoV-2 lineages used in this study were lineage B (Genbank access MT126808.1), Gamma variant (GISAID: EPI_ISL_2499748), and Delta variant (GISAID: EPI_ISL_2965577). Viral stocks were propagated in Vero E6 cells (ATCC CRL-1586) cultured in Dulbecco’s Modified Eagle’s Medium (DMEM) (Vitrocell, Brazil) containing 1% (*v*/*v*) penicillin/streptomycin and 2% (*v*/*v*) Fetal Bovine Serum (FBS) at 37 °C and 5% CO_2_ atmosphere. After 2 days, cell culture supernatant was collected, clarified by low-speed centrifugation, and stored at −80 °C until usage. Viral titration was performed by plaque-forming units (PFU) assay as previously described [13].

### 2.5. Plaque Reduction Neutralization Test 

The neutralizing antibody activity of serum samples was evaluated by the plaque reduction neutralization test (PRNT). Briefly, one day before infection, 10^5^ Vero E6 cells were plated per well on a 48 wells plate in DMEM containing 10% FBS and cultivated overnight at 37 °C and 5% CO_2_. The serum samples were heat-inactivated at 56 °C for 1 h. Then, serial dilutions were prepared from 1:5 to 1:160 in DMEM and mixed with 10^2^ PFUs of SARS-CoV-2. As a positive control, media only was used instead of sera samples. To allow antibody–virus complex formation, the mixture was incubated for 1 h at 37 °C. Then, cell culture supernatant was removed from the plates and the virus-serum mixture inoculated in duplicates onto cells monolayers, incubating for 1 h under gently horizontal agitation. Then, the residual viral inoculum was removed, and 500 µL of pre-warmed overlay medium (DMEM with 2% carboxymethylcellulose and 2% FBS) was added per well. The plates were incubated for 5 days at 37 °C and 5% CO_2_ to allow plaque formation. The cells were fixed with 4% formaldehyde solution and stained with 1% Naphthol blue-black (Sigma-Aldrich, USA) solution. Neutralization activity was determined by reduction in plaque formation when compared to the positive control. The area under the curve (AUC) value was determined using Prism v.8.0.2 (GraphPad, San Diego, CA, USA).

## 3. Results and Discussion

**Figure 1:**
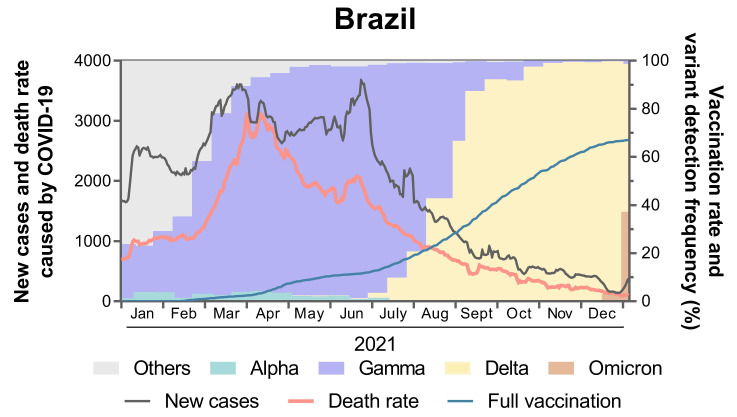
Epidemiological overview of SARS-CoV-2 lineages between 1st January to 31st December 2021 in Brazil. The black line indicates new cases of COVID-19 (7-day rolling average) per 10,000,000 residents and the red line indicates the daily death rate. The blue line indicates the percentage of fully vaccinated population and the colored bars indicate the prevalence of SARS-CoV-2 lineages. The raw data are available in Supplementary Tables S3 and S4.

The constant spread and evolution of SARS-CoV-2 have driven the emergence of the VOCs, which represent a threat to the global health system. The Delta variant was first detected in late 2020 in India, and since then it has spread to 182 countries, replacing other dominant lineages, including other VOCs in several countries, such as in the UK, USA, and Brazil (Figure 1) [14]. Although most Delta variant strains lack common mutations associated with neutralization antibodies escape (i.e., L18F, E484K, and N501Y), some studies have reported neutralization antibody resistance of Delta variant to sera of individuals vaccinated by BNT162b2 mRNA (Pfizer-BioNTech), mRNA-1273 (Moderna), ChAdOx1 (AstraZeneca), and Ad26.COV2.S (Janssen) vaccines [8,15,16,17]. In addition, convalescent sera from individuals infected by SARS-CoV-2 demonstrated reduced neutralization of Delta variant when compared to Alpha variant [17]. In our study, we investigated the neutralizing response induced by CoronaVac (Sinovac) and ChAdOx1 (AstraZeneca) vaccination and by natural infection caused by non-VOCs and the Gamma variant against authentic non-VOC isolate (PANGO lineage: B) and the Gamma and Delta variants. 

We performed neutralization assays with sera samples from 15 and 14 individuals vaccinated with CoronaVac and ChAdOx1, respectively; all collected between 3 to 7 weeks after the second dose. In addition, we tested serum samples from 18 convalescent individuals infected with the Gamma variant between February 03 and March 24 of 2021, and from 15 individuals infected between March 19 and May 26 of 2020, before the emergence of VOCs. All samples were tested against Gamma, Delta, and non-VOC isolate, previously confirmed by whole-genome sequencing. 

**Figure 2:**
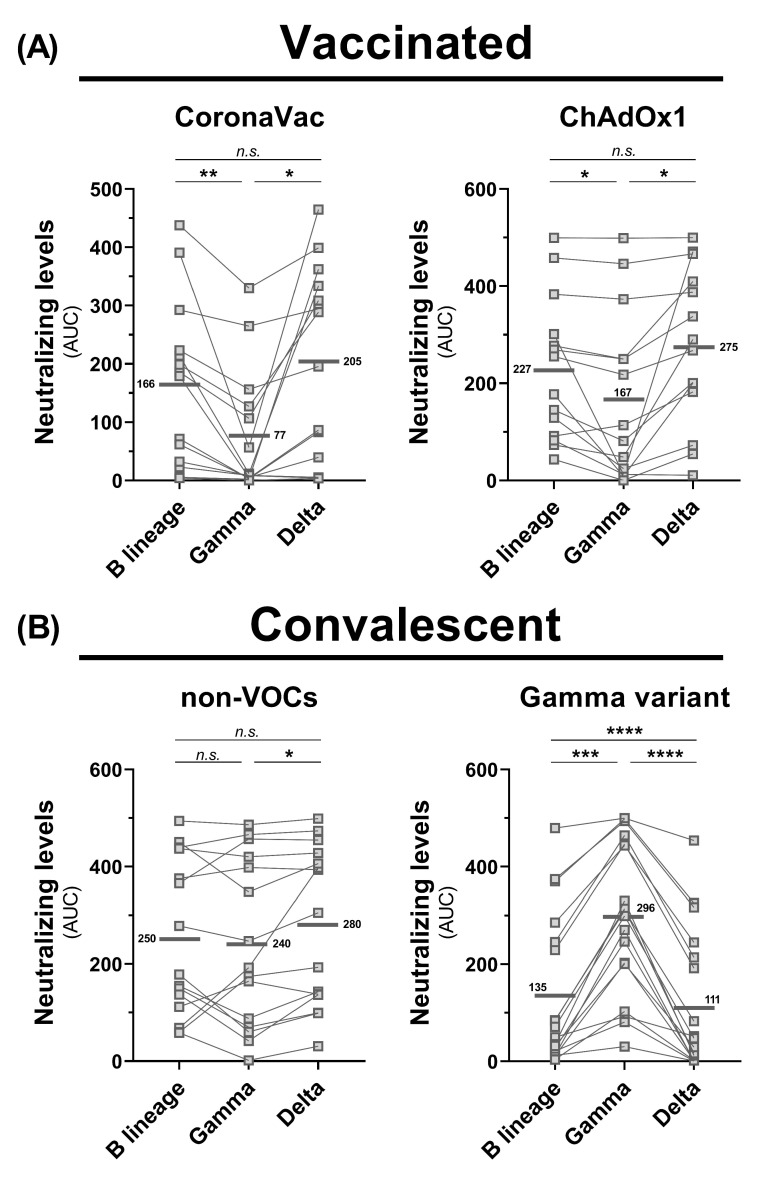
Serum neutralization assays for SARS-CoV-2 Delta variant, Gamma variant, and B lineage isolates. The assays were performed with live viruses from B lineage, Gamma variant, and Delta variant. Each data point represents the area under the curve (AUC) result obtained by plaque reduction neutralization testing (PRNT) using serially diluted sera from 1:5 to 1:160. The horizontal bars and numbers next to them indicated the average AUC per group. Panel A shows the serum neutralization results of individuals vaccinated by CoronaVac (*n* = 15) and ChAdOx1 (*n* = 14). Panel B shows the serum neutralization results of convalescent individuals infected by non-VOCs (*n* = 15) and by Gamma variant (*n* = 18). Statistical analysis was conducted by one-way ANOVA with matched pairs followed by Tukey’s post hoc (*, *p* < 0.05; **, *p* < 0.01; ***, *p* < 0.001; **** *p* < 0.0001; n.s., non-significant). Data information regarding the vaccinated donors and convalescent donors (sex, age, sampling date, and infection or vaccination dates) are summarized in Supplementary Tables S1 and S2.

We found that sera collected from individuals vaccinated with two doses of CoronaVac demonstrated 2.65 and 2.15 times higher neutralizing activities against Delta variant and B lineage isolates, respectively, than to the Gamma variant isolate (*p* < 0.05). Similarly, individuals vaccinated by ChAdOx1 demonstrated 1.65 and 1.36 times higher neutralizing capacity to Delta variant and B lineage isolates (*p* < 0.05), respectively, than to the Gamma variant isolate (Figure 2A). The enhanced resistance of the Gamma variant to neutralization by either convalescent or post-vaccination immune sera has been previously described [18,19]. In our study, sera from individuals previously infected by non-VOCs had 1.17 times higher neutralizing capacity to the Delta variant than to the Gamma variant (*p* < 0.05). Interestingly, these sera presented an approximately equivalent neutralization efficacy for both, B lineage and Delta variant. The antibody neutralization titers detected on sera from convalescent individuals recovered from a Gamma variant infection were 2.66 and 2.19 times lower to the Delta variant and B lineage (*p* < 0.0001), respectively, than to the Gamma variant isolate. Moreover, the neutralization capacity of the Delta variant was 0.82 times lower than that observed for the B lineage isolate (*p* < 0.0001) (Figure 2B). 

These findings indicate that the neutralizing capacities of antibodies elicited by vaccination with CoronaVac or ChAdOx1, and by infection with non-VOCs are not compromised against the Delta variant. However, we observed a significant decrease in serum neutralizing capacity from Gamma convalescent individuals against the Delta variant and B lineage isolates. Although the presence of neutralizing antibodies may correlate with protection, the impact of this data is challenging to predict due to multiple variables, such as the host genetic background, cellular immune response, and pathogenic cytotoxicity mediated by non-neutralizing antibodies [20,21]. Therefore, our data highlights the importance of naturally acquired immunity and continuous vaccination against the SARS-CoV-2 Delta variant. 

## Data Availability

The data generated in this study is present on the main text or in the Supplementary Materials.

## Supplementary Materials

The following are available online at https://www.mdpi.com/article/10.3390/v14020305/s1, Table S1. CoronaVac and ChAdOx1 vaccinated sera used in this study. Table S1. CoronaVac and ChAdOx1 vaccinated sera used in this study. Table S2. Convalescent sera used in this study. Table S3. SARS-CoV-2 variants detection frequency from January to December 2021 in Brazil. Table S4. Daily new cases, death rate, and percentage of full-vaccinated population in Brazil in 2021.
