# Supplementary material for: CoronaVac and ChAdOx1 Vaccination and Gamma Infection Elicited Neutralizing Antibodies against the SARS-CoV-2 Delta Variant
Source: Viruses. 2022 Feb 1;14(2):305. doi: 10.3390/v14020305 (PMC8880081; doi:10.3390/v14020305)
Supplement: Supplementary file 1 [file viruses-14-00305-s001.zip › viruses-1557200-supplementary.pdf]

Supplementary Material

Table of Contents

Supplementary Table S1. CoronaVac and ChAdOx1 vaccinated sera used in this study................2

Supplementary Table S2. Convalescent sera used in this study...3

Supplementary Table S3. SARS-CoV-2 variants detection frequency from January to December 2021 in Brazil...4

Supplementary Table S4. Daily new cases, death rate, and percentage of full-vaccinated population in Brazil in 2021..5

Supplementary Table S1. CoronaVac and ChAdOx1 vaccinated sera used in this study.

	Sample ID	Sex	Age	Date of 2nd vaccination (MM/DD/YY)	Serum collection (MM/DD/YY)	Neutralizing antibody capacity (AUC)		
						B lineage	Gamma	Delta
CoronaVac	113	Female	60	02/10/21	03/14/21	214.2	11.5	333.9
	115	Female	38	02/09/21	03/15/21	437.9	330.0	399.1
	117	Female	43	02/09/21	03/16/21	390.8	56.6	464.7
	141	Female	57	02/23/21	03/17/21	185.8	106.6	308.5
	143	Female	38	02/12/21	03/17/21	292.5	264.8	294.7
	150	Female	35	02/22/21	03/17/21	179.2	5.1	39.7
	153	Female	35	02/22/21	03/17/21	198.7	127.1	288.7
	154	Female	45	02/18/21	03/17/21	223.3	156.3	195.4
	160	Female	42	02/17/21	03/17/21	5.4	2.1	362.3
	171	Female	35	02/10/21	03/17/21	71.6	3.0	82.8
	173	Male	53	02/22/21	03/17/21	22.9	8.8	5.3
	182	Female	63	02/22/21	03/17/21	4.2	0.0	3.5
	184	Female	40	02/19/21	03/17/21	32.4	8.3	4.4
	191	Female	41	02/22/21	03/17/21	61.8	4.0	86.5
					**Geometric mean**	**165.8**	**77.4**	**205.0**
ChAdOx1	123	Female	64	02/09/21	03/05/21	277.2	250.0	409.6
	125	Female	28	02/15/21	03/05/21	269.0	250.0	337.6
	126	Male	55	02/12/21	03/05/21	301.4	12.6	471.2
	127	Female	47	02/12/21	03/05/21	177.4	8.6	200.6
	131	Female	60	02/09/21	03/08/21	383.2	373.1	387.4
	135	Female	34	02/11/21	03/08/21	255.3	217.7	267.4
	138	Female	44	02/09/21	03/11/21	73.1	48.1	290.2
	147	Female	55	02/02/21	03/17/21	91.4	114.0	182.3
	149	Female	37	02/01/21	03/17/21	458.2	446.0	466.6
	157	Female	37	02/02/21	03/17/21	499.5	498.9	499.8
	163	Female	50	02/01/21	03/17/21	145.2	81.4	201.8
	167	Female	39	02/01/21	03/17/21	82.7	12.6	10.8
	168	Female	37	01/29/21	03/17/21	43.4	0.3	54.5
	189	Male	52	02/01/21	03/17/21	129.0	24.5	72.6
					**Geometric mean**	**227.6**	**167.0**	**275.2**

Supplementary Table S2. Convalescent sera used in this study.

	Sample ID	Sex	Age	Disease severity	Date of test (MM/DD/YY)	RT-PCR (ΔCT)	Serum collection (MM/DD/YY)	Neutralizing antibody capacity (AUC)		
								B lineage	Gamma	Delta
Non-VOCs infected	1HC	Male	64	Severe	03/19/20	23,20	03/06/21	278.3	247.3	305.6
	2HC	Male	32	Severe	04/17/20	24,22	03/06/21	375.7	398.2	393.4
	3HC	Female	68	Severe	05/15/20	24,53	03/06/21	68.3	192.5	397.6
	4HC	Female	39	Severe	05/15/20	25,10	03/06/21	154.8	87.7	143.0
	5HC	Male	33	Severe	04/01/20	20,22	03/06/21	59.5	174.3	193.0
	6HC	Male	42	Severe	05/09/20	19,98	03/06/21	450.2	347.9	406.0
	7HC	Male	33	Severe	05/06/20	21,80	03/06/21	365.7	456.6	454.4
	8HC	Male	67	Severe	05/06/20	22,00	03/06/21	439.5	465.7	473.4
	9HC	Male	53	Severe	04/25/20	24,11	03/06/21	494.0	486.2	498.6
	10HC	Male	75	Severe	05/15/20	22,34	03/06/21	149.8	70.0	99.4
	11HC	Male	60	Severe	04/23/20	22,54	03/06/21	436.9	420.0	427.9
	12HC	Male	62	Severe	05/12/20	21,89	03/06/21	111.9	164.3	137.7
	13HC	Female	47	Severe	05/08/20	20,89	03/06/21	136.9	41.4	136.5
	14HC	Female	38	Severe	04/23/20	20,20	03/06/21	178.3	60.2	98.7
	15HC	Male	34	Severe	04/26/20	20,55	03/06/21	58.6	1.5	30.8
							**Geometric mean**	250.6	240.9	279.7
Gamma infected	1	Male	43	Mild	04/02/2021	22,6	12/05/2021	229.1	446.6	191.8
	2	Male	36	Mild	09/02/2021	20,27	20/05/2021	245.5	464.5	213.7
	3	Male	39	Mild	09/02/2021	16,47	20/05/2021	370.0	498.4	325.5
	4	Female	27	Mild	14/02/2021	22,21	14/05/2021	49.8	92.4	51.6
	5	Female	25	Mild	26/02/2021	27,52	20/05/2021	11.2	202.6	2.1
	6	Male	23	Mild	05/03/2021	24,66	20/05/2021	84.8	310.3	82.7
	7	Female	19	Mild	29/03/2021	21,91	18/05/2021	35.2	330.0	11.9
	8	Female	45	Mild	18/03/2021	23,08	18/05/2021	83.6	313.9	16.8
	9	Male	24	Mild	24/03/2021	23,56	18/05/2021	71.0	299.0	47.6
	10	Female	53	Mild	19/03/2021	21,04	20/05/2021	374.5	493.7	316.5
	11	Female	23	Mild	03/02/2021	25,65	12/05/2021	479.3	499.7	454.0
	12	Female	44	Mild	05/02/2021	23,55	12/05/2021	285.5	443.7	244.7
	13	Female	45	Mild	11/02/2021	26,11	20/05/2021	12.5	270.5	0.0
	14	Female	39	Mild	15/02/2021	22,85	20/05/2021	12.5	30.5	1.0
	15	Female	40	Mild	09/02/2021	22,1	20/05/2021	21.1	81.0	0.6
	16	Female	48	Mild	15/02/2021	24,93	20/05/2021	26.4	246.5	1.9
	17	Male	29	Mild	11/02/2021	24,25	20/05/2021	31.9	200.5	29.4
	18	Female	48	Mild	17/02/2021	22,87	20/05/2021	3.8	102.5	1.9
							**Geometric mean**	134.9	295.9	110.8

Supplementary Table S3. SARS-CoV-2 variants detection frequency from January to December 2021 in Brazil.

Date (MM/DD/YY)	SARS-CoV-2 variants detection frequency (%)				
	Alpha	Gamma	Delta	Omicron	Others
01/01/21	1.28	22.59	0	0	76.25
01/11/21	3.89	19.26	0	0	77
01/25/21	3.77	25.47	0	0	70.9
02/08/21	1.48	33.86	0	0	64.8
02/22/21	3.13	55.15	0	0	41.82
03/08/21	2.6	75.53	0	0	22
03/22/21	3.94	85.52	0	0	10.64
04/05/21	4.29	88.83	0	0	6.98
04/19/21	3.54	91.34	0	0	5.12
05/03/21	2.31	94.88	0.25	0	2.66
05/17/21	2.13	95.77	0.3	0	1.91
05/31/21	2.41	95.14	0.12	0	2.33
06/14/21	0.98	96.32	0.32	0	2.5
06/28/21	1.46	94.79	2.06	0	1.74
07/12/21	0.28	89.09	9.58	0	1.08
07/26/21	0.36	78.37	20.36	0	1.01
08/09/21	0.08	56.12	42.81	0	1.09
08/28/21	0.09	32.69	66.59	0	0.73
09/06/21	0.08	12.43	87.29	0	0.2
09/20/21	0	7.2	92.29	0	0.51
10/04/21	0	7.89	91.77	0	0.44
10/18/21	0	2.47	97.53	0	0
11/01/21	0	0.98	99.02	0	0
11/15/21	0	0.46	99.54	0	0
11/29/21	0	0.6	99.26	0.14	0
12/13/21	0	0.18	96.86	2.96	0
12/27/21	0	1.32	61.49	37.19	0

Supplementary Table S4. Daily new cases, death rate, and percentage of full-vaccinated population in Brazil in 2021.

Date (MM/DD/YY)	Daily 7-day rolling average cases per 10.000.000 habitants	Daily death rate	Full-vaccinated population (%)
01/01/21	1682	703	0
01/02/21	1667	703	0
01/03/21	1665	699	0
01/04/21	1649	708	0
01/05/21	1660	727	0
01/06/21	1706	729	0
01/07/21	1930	801	0
01/08/21	2174	888	0
01/09/21	2415	991	0
01/10/21	2494	1016	0
01/11/21	2532	1001	0
01/12/21	2575	992	0
01/13/21	2557	997	0
01/14/21	2426	945	0
01/15/21	2489	953	0
01/16/21	2536	953	0
01/17/21	2552	962	0
01/18/21	2540	960	0
01/19/21	2525	965	0
01/20/21	2556	984	0
01/21/21	2493	1011	0
01/22/21	2410	1002	0
01/23/21	2409	1021	0
01/24/21	2389	1027	0
01/25/21	2400	1058	0
01/26/21	2397	1057	0
01/27/21	2398	1048	0
01/28/21	2411	1061	0
01/29/21	2430	1064	0
01/30/21	2395	1070	0
01/31/21	2391	1067	0
02/01/21	2378	1057	0
02/02/21	2342	1072	0
02/03/21	2273	1053	0
02/04/21	2248	1029	0
02/05/21	2196	1053	0
02/06/21	2151	1016	0.01
02/07/21	2139	1005	0.01
02/08/21	2140	1019	0.02
02/09/21	2106	1027	0.02
02/10/21	2151	1050	0.04
02/11/21	2127	1074	0.05
02/12/21	2109	1069	0.08
02/13/21	2099	1082	0.09

02/14/21	2130	1105	0.1
02/15/21	2111	1090	0.13
02/16/21	2205	1058	0.26
02/17/21	2112	1033	0.34
02/18/21	2112	1033	0.42
02/19/21	2133	1049	0.49
02/20/21	2183	1049	0.53
02/21/21	2175	1043	0.55
02/22/21	2215	1057	0.6
02/23/21	2204	1095	0.67
02/24/21	2325	1132	0.75
02/25/21	2424	1151	0.82
02/26/21	2499	1153	0.88
02/27/21	2539	1185	0.89
02/28/21	2565	1208	0.9
03/01/21	2622	1225	0.95
03/02/21	2596	1277	1.01
03/03/21	2647	1334	1.08
03/04/21	2698	1368	1.15
03/05/21	2759	1421	1.22
03/06/21	2816	1455	1.26
03/07/21	3138	1501	1.27
03/08/21	3113	1543	1.33
03/09/21	3190	1576	1.4
03/10/21	3247	1652	1.48
03/11/21	3254	1707	1.55
03/12/21	3321	1766	1.62
03/13/21	3344	1832	1.66
03/14/21	3089	1828	1.67
03/15/21	3140	1858	1.72
03/16/21	3236	1987	1.77
03/17/21	3284	2027	1.83
03/18/21	3357	2102	1.88
03/19/21	3395	2179	1.92
03/20/21	3414	2238	1.94
03/21/21	3441	2263	1.94
03/22/21	3518	2301	1.99
03/23/21	3516	2344	2.03
03/24/21	3531	2295	2.08
03/25/21	3600	2285	2.13
03/26/21	3547	2407	2.17
03/27/21	3604	2555	2.19
03/28/21	3601	2603	2.2
03/29/21	3511	2662	2.25
03/30/21	3520	2734	2.32
03/31/21	3505	2964	2.38
04/01/21	3435	3107	2.45
04/02/21	3362	3006	2.47

04/03/21	3079	2791	2.5
04/04/21	2981	2743	2.52
04/05/21	2982	2728	2.62
04/06/21	2973	2787	2.75
04/07/21	2973	2751	2.85
04/08/21	2978	2822	2.99
04/09/21	3103	2957	3.2
04/10/21	3285	3024	3.27
04/11/21	3330	3112	3.3
04/12/21	3310	3084	3.46
04/13/21	3294	3039	3.62
04/14/21	3200	3007	3.81
04/15/21	3084	2913	4.01
04/16/21	3059	2854	4.29
04/17/21	3072	2925	4.45
04/18/21	3070	2877	4.49
04/19/21	3049	2884	4.77
04/20/21	2982	2825	5.01
04/21/21	2978	2790	5.12
04/22/21	2824	2584	5.33
04/23/21	2723	2542	5.74
04/24/21	2691	2526	5.85
04/25/21	2657	2499	5.88
04/26/21	2629	2445	6.16
04/27/21	2651	2407	6.55
04/28/21	2673	2381	6.84
04/29/21	2809	2521	7.09
04/30/21	2828	2495	7.34
05/01/21	2791	2420	7.4
05/02/21	2762	2410	7.42
05/03/21	2806	2374	7.62
05/04/21	2764	2359	7.82
05/05/21	2758	2325	7.98
05/06/21	2782	2254	8.11
05/07/21	2815	2158	8.24
05/08/21	2838	2135	8.28
05/09/21	2861	2086	8.29
05/10/21	2843	2084	8.45
05/11/21	2847	1979	8.59
05/12/21	2846	1952	8.73
05/13/21	2897	1915	8.79
05/14/21	2904	1915	8.88
05/15/21	2942	1903	8.92
05/16/21	2974	1923	8.96
05/17/21	2969	1920	9.08
05/18/21	3001	1949	9.21
05/19/21	3028	1937	9.31
05/20/21	3050	1971	9.44

05/21/21	3041	1962	9.56
05/22/21	3045	1923	9.62
05/23/21	3051	1898	9.72
05/24/21	3064	1866	9.86
05/25/21	3059	1828	10
05/26/21	3059	1823	10.1
05/27/21	2933	1764	10.2
05/28/21	2781	1807	10.32
05/29/21	2834	1838	10.35
05/30/21	2854	1841	10.37
05/31/21	2848	1856	10.44
06/01/21	2881	1878	10.54
06/02/21	2988	1884	10.67
06/03/21	3065	1857	10.71
06/04/21	2993	1681	10.75
06/05/21	2898	1642	10.79
06/06/21	2891	1638	10.81
06/07/21	2912	1664	10.86
06/08/21	2757	1709	10.94
06/09/21	2681	1692	11
06/10/21	2782	1776	11.06
06/11/21	3070	1917	11.12
06/12/21	3151	1961	11.13
06/13/21	3128	1995	11.14
06/14/21	3148	1977	11.18
06/15/21	3358	1982	11.23
06/16/21	3369	2026	11.27
06/17/21	3271	1999	11.33
06/18/21	3332	2042	11.39
06/19/21	3359	2075	11.43
06/20/21	3410	2062	11.44
06/21/21	3428	2056	11.49
06/22/21	3676	1973	11.56
06/23/21	3615	1911	11.63
06/24/21	3616	1871	11.78
06/25/21	3480	1804	11.87
06/26/21	3365	1706	11.93
06/27/21	3280	1662	11.95
06/28/21	3191	1624	12.09
06/29/21	2802	1600	12.31
06/30/21	2600	1571	12.45
07/01/21	2497	1556	12.59
07/02/21	2420	1537	12.74
07/03/21	2374	1554	12.81
07/04/21	2330	1562	12.85
07/05/21	2303	1572	13.02
07/06/21	2274	1547	13.23
07/07/21	2321	1478	13.52

07/08/21	2274	1449	13.81
07/09/21	2240	1391	14.04
07/10/21	2176	1323	14.2
07/11/21	2134	1294	14.27
07/12/21	2076	1293	14.49
07/13/21	1988	1278	14.75
07/14/21	2001	1271	15.06
07/15/21	2003	1254	15.34
07/16/21	1899	1243	15.64
07/17/21	1932	1259	15.8
07/18/21	1916	1247	15.84
07/19/21	1899	1230	16.07
07/20/21	1783	1190	16.38
07/21/21	1781	1171	16.73
07/22/21	1743	1146	17.14
07/23/21	2172	1132	17.37
07/24/21	2076	1104	17.51
07/25/21	2086	1104	17.56
07/26/21	2117	1098	17.81
07/27/21	2200	1085	18.11
07/28/21	2141	1081	18.51
07/29/21	2092	1068	18.86
07/30/21	1647	1010	19.21
07/31/21	1650	992	19.4
08/01/21	1664	982	19.44
08/02/21	1647	971	19.7
08/03/21	1573	963	20.01
08/04/21	1529	920	20.45
08/05/21	1517	890	20.75
08/06/21	1532	900	21.07
08/07/21	1562	907	21.21
08/08/21	1519	904	21.28
08/09/21	1496	899	21.57
08/10/21	1516	903	21.95
08/11/21	1482	899	22.26
08/12/21	1458	883	22.61
08/13/21	1393	874	22.97
08/14/21	1325	861	23.18
08/15/21	1324	861	23.23
08/16/21	1342	848	23.65
08/17/21	1366	823	24.19
08/18/21	1397	815	24.59
08/19/21	1398	816	25.09
08/20/21	1406	817	25.5
08/21/21	1380	774	25.71
08/22/21	1381	766	25.78
08/23/21	1364	766	26.17
08/24/21	1316	732	26.64

08/25/21	1248	718	27.03
08/26/21	1214	696	27.48
08/27/21	1171	685	27.92
08/28/21	1155	688	28.23
08/29/21	1137	680	28.35
08/30/21	1118	666	28.69
08/31/21	1085	669	29.3
09/01/21	1057	642	29.79
09/02/21	1031	629	30.33
09/03/21	1003	618	30.89
09/04/21	985	619	31.2
09/05/21	995	618	31.3
09/06/21	977	604	31.64
09/07/21	889	525	31.79
09/08/21	812	461	32.31
09/09/21	850	467	32.99
09/10/21	803	452	33.73
09/11/21	759	457	34.14
09/12/21	727	461	34.28
09/13/21	717	470	34.76
09/14/21	716	520	35.43
09/15/21	710	599	35.98
09/16/21	728	576	36.44
09/17/21	854	576	37.17
09/18/21	777	564	37.5
09/19/21	771	557	37.61
09/20/21	775	554	37.97
09/21/21	787	553	38.54
09/22/21	916	534	39.09
09/23/21	846	529	39.66
09/24/21	734	535	40.24
09/25/21	827	529	40.56
09/26/21	825	531	40.71
09/27/21	825	521	41.04
09/28/21	835	542	41.64
09/29/21	730	544	42.18
09/30/21	711	541	42.73
10/01/21	707	514	43.35
10/02/21	697	501	43.67
10/03/21	699	496	43.77
10/04/21	713	497	44.08
10/05/21	748	478	44.51
10/06/21	759	462	45.02
10/07/21	710	437	45.51
10/08/21	714	457	46.01
10/09/21	730	451	46.3
10/10/21	725	440	46.51
10/11/21	695	441	46.77

10/12/21	596	368	46.88
10/13/21	528	316	47.13
10/14/21	532	337	47.79
10/15/21	504	319	48.38
10/16/21	470	326	48.75
10/17/21	455	323	48.89
10/18/21	471	322	49.17
10/19/21	510	351	49.7
10/20/21	559	379	50.27
10/21/21	571	364	50.56
10/22/21	565	355	50.93
10/23/21	579	339	51.13
10/24/21	568	337	51.38
10/25/21	558	338	52.13
10/26/21	559	342	52.61
10/27/21	568	347	53.11
10/28/21	546	333	53.56
10/29/21	541	328	54
10/30/21	529	309	54.16
10/31/21	539	311	54.26
11/01/21	518	296	54.34
11/02/21	468	260	54.39
11/03/21	463	225	54.68
11/04/21	460	234	55.11
11/05/21	462	230	55.72
11/06/21	470	242	55.92
11/07/21	467	233	56.03
11/08/21	482	236	56.49
11/09/21	521	244	56.89
11/10/21	512	255	57.31
11/11/21	528	227	57.76
11/12/21	538	262	58.14
11/13/21	526	262	58.35
11/14/21	517	261	58.47
11/15/21	490	254	58.63
11/16/21	455	245	58.84
11/17/21	442	260	59.33
11/18/21	414	265	59.65
11/19/21	400	210	59.99
11/20/21	392	196	60.21
11/21/21	397	200	60.38
11/22/21	407	208	60.67
11/23/21	446	230	61
11/24/21	440	215	61.32
11/25/21	446	217	61.55
11/26/21	436	227	61.87
11/27/21	437	230	62
11/28/21	426	227	62.1

11/29/21	429	226	62.17
11/30/21	414	231	62.58
12/01/21	414	230	62.92
12/02/21	412	217	63.24
12/03/21	410	207	63.76
12/04/21	404	195	63.94
12/05/21	412	194	63.98
12/06/21	422	196	64.19
12/07/21	421	187	64.44
12/08/21	407	183	64.6
12/09/21	386	183	64.92
12/10/21	362	183	65.11
12/11/21	330	169	65.14
12/12/21	310	171	65.15
12/13/21	288	158	65.32
12/14/21	250	137	65.49
12/15/21	216	146	65.84
12/16/21	182	135	65.97
12/17/21	164	130	66.08
12/18/21	162	142	66.11
12/19/21	161	139	66.12
12/20/21	160	144	66.22
12/21/21	156	138	66.32
12/22/21	143	116	66.53
12/23/21	144	117	66.55
12/24/21	139	112	66.59
12/25/21	148	96	66.61
12/26/21	172	95	66.64
12/27/21	200	93	66.71
12/28/21	236	108	66.81
12/29/21	279	104	66.96
12/30/21	339	108	67.01
12/31/21	382	97	67.03
